# Co-localization of GSTP1 and JNK in transitional cell carcinoma of urinary bladder

**DOI:** 10.1590/s1415-47572010005000063

**Published:** 2010-09-01

**Authors:** Marija Pljesa-Ercegovac, Ana Savic-Radojevic, Tamara Kravic-Stevovic, Vladimir Bumbasirevic, Jasmina Mimic-Oka, Tatjana Simic

**Affiliations:** 1 Faculty of Medicine, Institute of Medical and Clinical Biochemistry, University of Belgrade, BelgradeSerbia; 2 Faculty of Medicine, Institute of Histology and Embryology, University of Belgrade, BelgradeSerbia

**Keywords:** glutathione S-transferase, JNK, TCC, co-localization

## Abstract

Transitional cell carcinoma (TCC) of urinary bladder belongs to glutathione S-transferase P1 (GSTP1) overexpressing tumors. Upregulated GSTP1 in TCC is related to apoptosis inhibition. This antiapoptotic effects of GSTP1 might be mediated through protein:protein interaction with c-Jun NH_2_ -terminal kinase (JNK). Herein, we analyzed whether a direct link between GSTP1 and JNK exists in TCC. The presence of GSTP1/JNK complexes was analyzed by immunoprecipitation and Western blotting in 20 TCC specimens, obtained after surgery. Co-localization of GSTP1 and JNK was also investigated in the 5637 TCC cell line by immunofluorescence confocal microscopy. By means of immunoprecipitation we show for the first time the presence of GSTP1/JNK complexes in all TCC samples studied. A co-localization of GSTP1 and JNK was also demonstrated in the 5637 TCC cell line by means of confocal microscopy. Protein-protein interactions, together with co-localization between GSTP1 and JNK provide evidence that GSTP1 most probably inhibits apoptosis in TCC cells by non-covalent binding to JNK.

The glutathione transferases are a multigene family of isozymes that catalyze the nucleophilic attack of the sulfur atom of glutathione on electrophilic groups of substrate molecules (Hayes and Strange, 2000). Glutathione transferase P1 (GSTP1) is the most prevalent in mammalian cells (Townsend and Tew, 2003). GSTP1 is overexpressed in many tumors, including transitional cell carcinoma (TCC) of urinary bladder, where its activity and expression correlate with tumor stage and grade (Berendsen *et al.*, 1997; Townsend and Tew, 2003; Simic *et al.*, 2005). Because of the defined role of GST in drug metabolism, elevated expression of GSTP1 in tumors has been frequently associated with detoxification reactions. Nevertheless, GSTP1 overexpression has been found in drug resistant cells, even in instances where there is no evidence that the selecting drug is a substrate for GSTP1 (Gate and Tew, 2001; Townsend and Tew, 2003). Recently, a new insight into a functional link between upregulated GSTP1 and the malignant phenotype has been suggested from growing evidence that GSTs are also involved in the regulation of stress signaling and resistance to apoptosis by mechanisms independent of their catalytic activity; this regulatory role depending on cellular redox status (Wang *et al.*, 2001; Adler and Pincus, 2004; Simic *et al.*, 2009). In TCC, the oxidant-antioxidant balance favors the reduced state, as increased levels of glutathione, the major cellular nonprotein antioxidant, together with upregulated antioxidant enzymes have been observed in this setting (Yang *et al.*, 1997; Savic-Radojevic *et al.*, 2007). High intracellular thiol levels and the absence of oxidative stress promote the existence of GSTP1 in monomeric form, while catalitically active GSTP1 is normally dimerized. Redox-active monomeric GSTP1 subunits inhibit c-Jun NH_2_-terminal kinase (JNK), an enzyme that triggers the apoptotic cascade in several cancer cell lines (Wang *et al.*, 2001). From our point of view, high antioxidant capacity also promotes an antiapoptotic role of GSTP1 in TCC. In favor of such a hypothesis are recent data showing a significant negative correlation between GSTP1 and cleaved caspase 3 expression in human TCC specimens (Pljesa-Ercegovac *et al.*, 2009). Nevertheless, the direct link between GSTP1 and JNK in TCC still has to be confirmed. Herein, we analyzed the presence of GSTP1/JNK complexes in specimens of tumor tissue of the urinary bladder obtained from 20 patients with TCC after radical cystectomy, as well as in the 5637 TCC cell line. Specimens of tumor tissue were taken in the operating theatre in the presence of a clinical pathologist who performed the histopathology examination. All patients gave informed consent to enter the study. The ethics committee approved the use of human tissue for research.

Tumor samples were washed in cold saline, frozen in liquid nitrogen and stored at -80 °C until use. Immunoprecipitation experiments were performed using the primary antibody against JNK (Sigma-Aldrich, St. Louis, USA) and the Protein A-agarose (Roche Applied Science, Mannheim, Germany). Samples were homogenized with the lysis buffer provided by the manufacturer and centrifuged at 100.000 x g for 45 min at 4 °C. Cytosolic fractions of TCC specimens were incubated with 2 μL of anti-JNK antibody overnight at 4 °C. Immunoblots were then probed with the anti-GSTP1 antibody (Sigma-Aldrich, St. Louis, USA). JNK expression was determined on the same membrane after stripping off the immune complex for the detection of GSTP1. Immunoblot analysis showed an absence of non-specific binding of the JNK antibody to GSTP1. Control immunoprecipitations, that were performed in the absence of anti-JNK antibody, ruled out possible unspecific pull-down of GSTP1. Confocal microscopy on cells of the 5637 cell line was performed using anti-GSTP1 antibody followed by FITC-conjugated secondary antibody (Dako, Glostrup, Danmark), as well as anti-JNK antibody followed by TRITC-conjugated secondary antibody (Dako, Glostrup, Danmark). Coverslips were mounted with fluorescent mounting medium (Dako, Glostrup, Danmark), observed and photographed under confocal scanning microscope (Leica LCS). The specificity of the primary antibodies used was previously confirmed by Western blot analysis. Control experiments for non-specific binding were performed in parallel by omission of the primary antibody.

Immunoprecipitation, followed by Western blotting showed the presence of GSTP1/JNK complexes in all TCC samples studied (Figure 1A). Furthermore, co-localization of GSTP1 and JNK was confirmed in 5637 TCC cell line by means of immunofluorescence confocal microscopy (Figure 1B). These results are in agreement with data of Adler and Pincus (2004) who performed molecular dynamics investigations on the 3-dimensional structure of GSTP1, free and bound to an inhibitor, that blocks its ability to inhibit JNK activation (Adler and Pincus, 2004). Four putative domains are involved in the interaction between GSTP1 and JNK. Two of these are involved in GSTP1 binding to JNK, whereas the other two affect phosphorylation of JNK. The proposed mechanism by which GSTP1 inhibits activation of JNK is by either blocking phosphorylation of JNK or by promoting dephosphorylation of phosphorylated JNK (Adler and Pincus, 2004). In this manner, JNK is prevented from activating downstream targets in the apoptotic pathway, such as c- Jun. Our results support such a hypothesis, because the activated JNK signal was not detected in our immunoprecipitation experiments.

The presence of GSTP1/JNK complex has also been reported in human leukemia, hepatic carcinoma and neuroblastoma cells (Ricci *et al.*, 2005; Turella *et al.*, 2005; Cui *et al.*, 2008; Castro-Caldas *et al.*, 2009). Therefore, it is possible that in these cells GSTP1 may act as an inhibitor of apoptosis, controlling JNK catalytic activity. In addition to enhancement of tumor progression, upregulated GSTP1 expression in TCC might also limit the efficacy of chemotherapeutic agents that act by inducing apoptosis via the JNK pathway (Wang *et al.*, 2001; Townsend and Tew, 2003; Burg *et al.*, 2006; Lo and Ali-Osman, 2007). To date, several chemotherapeutic agents that are being used in various stages of TCC, such as mytomicine c, adriamycine and cysplatin, induce apoptosis in tumor cells via the JNK pathway. We believe that the presence of GSTP1 in complexes with JNK limits the efficacy of intravesicular and systemic chemotherapy in bladder cancer, affecting patients' survival. In order to improve the response to chemotherapy it seems reasonable that novel small-molecule GSTP1-targeted agents, which were developed to overcome resistance to treatment in ovarian, non-small-cell lung, breast, and colorectal cancers, can also be used to sensitize TCC tumor cells (Tew, 2005; Turella *et al.*, 2005; Vergote *et al.*, 2007).

**Figure 1:**
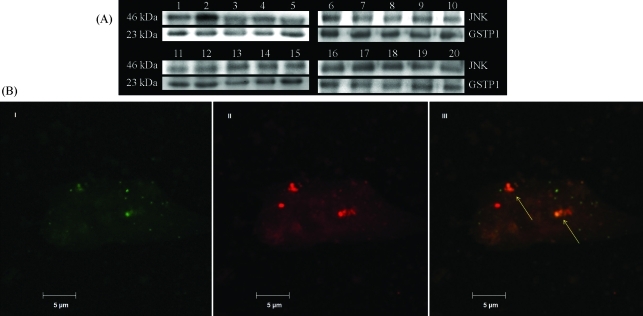
(A) Co-immunoprecipitation of GSTP1 with JNK in human TCC specimens. Immunoprecipitation was performed using protein A-agarose to retain JNK/GSTP1 complexes. Anti-JNK antibody and polyclonal anti-GSTP1 antibody were used for Western blot analysis. (B) Confocal microscopy images of human 5637 TCC cells after incubation with anti-GSTP1 antibody followed by FITC-conjugated secondary antibody (I); after incubation with anti-JNK antibody followed by TRITC-conjugated secondary antibody (II); Image showing co-localization of GSTP1 with JNK (III). Coverslips were mounted with fluorescent mounting medium, observed and photographed under confocal scanning microscope.

In conclusion, this study adds to the generality of the interaction between GSTP1 and JNK as a contributing factor to TCC phenotype and might have important implications in the treatment of these tumors.
